# Signatures of Selection in the Genomes of Chinese Chestnut (*Castanea mollissima* Blume): The Roots of Nut Tree Domestication

**DOI:** 10.3389/fpls.2018.00810

**Published:** 2018-06-25

**Authors:** Nicholas R. LaBonte, Peng Zhao, Keith Woeste

**Affiliations:** ^1^Department of Crop Sciences, University of Illinois Urbana-Champaign, Urbana, IL, United States; ^2^Key Laboratory of Resource Biology and Biotechnology in Western China, Ministry of Education, College of Life Sciences, Northwest University, Xi'an, China; ^3^Hardwood Tree Improvement and Regeneration Center, Northern Research Station, USDA Forest Service, West Lafayette, IN, United States

**Keywords:** chestnut, Fagaceae, crop domestication, Illumina sequencing, nut tree, pool-seq, selective sweep, woody perennial

## Abstract

Chestnuts (*Castanea*) are major nut crops in East Asia and southern Europe, and are unique among temperate nut crops in that the harvested seeds are starchy rather than oily. Chestnut species have been cultivated for three millennia or more in China, so it is likely that artificial selection has affected the genome of orchard-grown chestnuts. The genetics of Chinese chestnut (*Castanea mollissima* Blume) domestication are also of interest to breeders of hybrid American chestnut, especially if the low-growing, branching habit of Chinese chestnut, an impediment to American chestnut restoration, is partly the result of artificial selection. We resequenced genomes of wild and orchard-derived Chinese chestnuts and identified selective sweeps based on pooled whole-genome SNP datasets. We present candidate gene loci for chestnut domestication and discuss the potential phenotypic effects of candidate loci, some of which may be useful genes for chestnut improvement in Asia and North America. Selective sweeps included predicted genes potentially related to flower phenology and development, fruit maturation, and secondary metabolism, and included some genes homologous to domestication candidates in other woody plants.

## Introduction

Traits relevant to plant domestication show genomic evidence of selection in diverse crop species, including grains (Cockram et al., 2007), legumes (Kaga et al., 2008; Lam et al., 2010; Li et al., 2013; Schmutz et al., 2014), annual fruit-bearing crops such as tomato and squash (Lefebvre et al., 1998; Frary et al., 2000; Ronen et al., 2000; Vrebalov et al., 2002; Rao and Paran, 2003; Guo et al., 2013), and woody perennial fruit crops (Cao et al., 2014; Khan et al., 2014). Some traits, such as flowering time and plant architecture, show genomic evidence of selection in many crops (Mao et al., 2000; Clark et al., 2006; Paran and van der Knaap, 2007; Tan et al., 2008; Zhou et al., 2009; Li et al., 2013). Other traits, such as fruit quality (Qi et al., 2013; Khan et al., 2014; Qin et al., 2014) or seed size (Shomura et al., 2008; Wang et al., 2008), are associated with genomic evidence of selection only in specific types of crops. Signatures of selection in the genomes of woody perennial crops may be obscured by longer generation times and more widespread self-incompatibility (Cornille et al., 2012) than annual crop plants. Nevertheless, parts of the genomes of grape (Zhou et al., 2017), peach (Cao et al., 2014; Akagi et al., 2016), and apple (Khan et al., 2014; Duan et al., 2017) have been identified as candidates for selection during domestication.

Chestnut (primarily *Castanea mollissima*) was first deliberately cultivated as a food plant in China at least 2000 years before present (ybp) (Rutter et al., 1991; Wang, 2004), likely more recently than the domestication of apples (4,000 ybp: Cornille et al., 2012) or of peach and almond (5,000 ybp: Velasco et al., 2016). It is possible that humans began artificially selecting chestnuts earlier than 2,000 ybp: an increase in chestnut pollen, at the expense of conifers, is noted in the archaeological record of northwest China around 4,600 ybp, which coincides with the appearance of grain cultivation (Li et al., 2007). Today, chestnut is an economically valuable crop and China is the world's largest producer (Metaxas, 2013). Chestnut orchards in China include both seedling trees and grafted cultivars, mostly of *C. mollissima*, with some regional use of *C. henryi, C. crenata*, or interspecific hybrids (Wang, 2004). The timing of flower development, pollination, and fertilization of ovules is crucial for optimizing chestnut yield (Shi and Stoesser, 2005); self-pollination does not normally occur (Pereira-Lorenzo et al., 2016). Characteristics currently under selection in improvement programs for Chinese orchard chestnuts include attractive (shiny) appearance of nuts, early maturation and bearing, stable yield, high sugar content, pest and disease resistance, and adaptation to orchard environments that are hotter and drier than the mountains where most wild *C. mollissima* occur (Zhang et al., 2010). Shorter catkins are also desired (Huang et al., 2009), as are large seeds (~20 g) (Xu et al., 2010), especially for commercial paste-production cultivars, and a pellicle that is easy to peel (Takada et al., 2012). Post-harvest diseases that destroy chestnuts in storage are a major concern (Ma et al., 2000). A study in Japanese chestnut (Nishio et al., 2017) recently revealed quantitative trait loci associated with a set of traits including harvest date, nut weight, and pericarp splitting. Broad-sense heritability estimates for these traits ranged from 0.40 (nut weight) to 0.91 (harvest date) (Nishio et al., 2014).

Traits possibly under selection during chestnut domestication include the traits currently targeted for improvement, as well as others, including plant architecture. A small, branchy tree is more manageable in an orchard setting than a very tall one, especially in locales where chestnuts are picked by hand after climbing the tree (Rutter et al., 1991). Chinese chestnut in general has a shorter stature and less-pronounced apical dominance than the non-domesticated American chestnut (Clapper, 1954) which is a major consideration in the backcross blight resistance breeding program being carried out by the American Chestnut Foundation (Burnham et al., 1986). In forest settings, *C. mollissima* grow to 20–25 m in height (Fei et al., 2012), so the short stature of orchard trees may be, at least in part, an artificially selected trait. Chestnuts are highly perishable (Rutter et al., 1991) so genes related to pericarp thickness and wax coatings on the pericarp may be important if they confer improved storage qualities. Fruit quality genes, while they may not affect the flavor of the chestnut, could be under selection for human aesthetic preferences; Clapper (1954) noted variation in the color of Chinese chestnuts that was not seen in American chestnuts. Finally, although preference for large seeds varies across China (Wang, 2004; Yang et al., 2015), seed size is a likely cause for artificial selection in Chinese chestnut, especially for “processing” varieties intended for the industrial production of paste and flour (e.g., Xu et al., 2010).

In addition to differentiation between cultivated and wild Chinese chestnut, there is likely to be differential selection among regional subpopulations of wild trees: Chinese chestnut occupies a larger range than any other Asian or American species of *Castanea* (Fei et al., 2012). The natural selective pressure on Chinese chestnut populations is likely to vary considerably between its temperate, high-altitude habitat in the Qin Mountains (northwest China) and the subtropical provinces of Yunnan and Guizhou. Considerable rangewide genetic variation, at the whole-genome scale, has been identified in forest tree genomes, including poplar (Slavov et al., 2012) and whitebark pine (Syring et al., 2016). Genetic diversity of wild Chinese chestnut has been analyzed with varying results; southwest (Zhang and Liu, 1998) and northwest China (Shaanxi Province; Cheng et al., 2012) have been proposed as centers of genetic diversity for the species. Given its wide distribution, the census population size of Chinese chestnut is probably similar to the estimated 3-4 billion American chestnuts (*Castanea dentata*) that grew in eastern North America prior to the introduction of chestnut blight disease (Hebard, 2012); given its outcrossing habit, this likely corresponds to a very high effective population size in wild Chinese chestnut. While genetic diversity is higher in wild trees, it appears that a high level of genetic diversity has been maintained in orchard (domesticated) Chinese chestnuts (Pereira-Lorenzo et al., 2016), although the genetic diversity of new cultivars may be lower than traditional orchard trees (Ovesna et al., 2004).

Signatures of selection due to domestication are generally identified as regions of the genome where, using statistics related to nucleotide diversity and heterozygosity (Tajima's D, pi, *F*_ST_), reduction of allelic diversity in domesticated lineages, vs. wild lineages, is determined to be signficant (Teshima et al., 2006; Purugganan and Fuller, 2009); these regions may be called “selective sweeps.” Dozens or hundreds of relatively small genomic intervals may show evidence of a selective sweep in a domesticated plant genome. Given the large number of statistical tests, the likelihood that sweeps will be observed by chance alone (false positives) is high (Thornton and Jensen, 2007), although statistical methods for ameliorating this problem are available(Burger et al., 2008). Genes identified in domestication regions, if subsequent investigation confirms their predicted function and phenotypic effects, could be important for further improvement of Chinese and other chestnut species for orchard production.

We investigated the following questions:

1. Is genetic diversity on the genomic scale lower in orchard-derived Chinese chestnut than it is in wild Chinese chestnut?
2. What regions of the genome show evidence of selective sweeps in the genome of domesticated Chinese chestnut, and are these regions syntenic with regions under selection in other woody plants?
3. Do northern (Shaanxi Province) and southern (Yunnan and Guizhou) gene pools of wild Chinese chestnut present different signatures of selection?

To answer these questions, we utilized whole-genome resequencing with a pool-seq approach. Because we investigated genetic differentiation among different groups (pools) of trees rather than individual trees, it was feasible to estimate allele frequencies and genetic statistics (pi and Tajima's D) from pools of samples rather than individual genome sequences (Lynch et al., 2014) Pool-seq may reduce the precision of allele frequency estimates, but becausethere was no individual phenotype information (e.g. disease resistance, seed size) available for most of our samples, the potential gains from sequencing individuals was limited. Because the sequencing cost per individual was less, more individuals (a larger sample of the total genetic variation among wild and orchard trees) could be used to estimate population genetics statistics (Schlöetterer et al., 2014; Chen et al., 2016). We validated candidate loci for selection under domestication, identified by the pool-seq analysis, by analyzing nucleotide diversity statistics and heterozygosity of the same genomic regions in an independent sample of high-coverage genome sequences of 17 orchard-derived Chinese chestnut accessions.

## Materials and methods

### DNA samples

**Figure 1:**
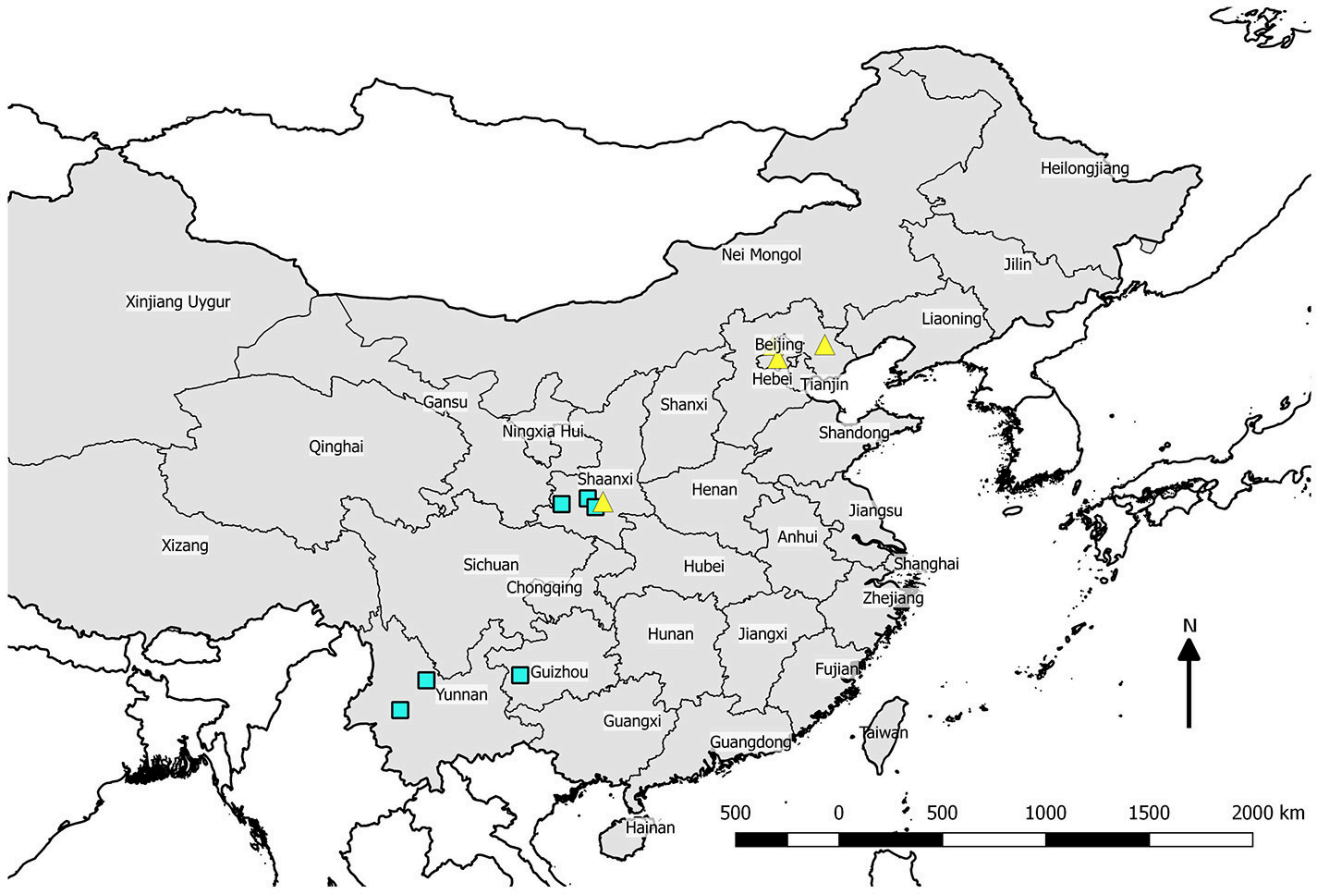
Map of the People's Republic of China showing locations from which wild trees were sampled (open squares) and the location of orchards sampled (filled triangles).

Leaf samples were collected in China during 2015, rapidly dried using desiccant beads, and mailed to Purdue University for DNA isolation in 2016 following applicable regulations for the importation of plant DNA samples. Trees classified as wild were sampled from natural montane forests where it is relatively unlikely that groves of chestnut represent escapes from cultivation (Figure 1). Orchard trees were sampled from orchard settings in northeast China where most commercial growing takes place (Table 1). The United States sample of orchard-derived Chinese chestnut was grown at Empire Chestnut Company, Carrollton, OH, from Beijing-area source material. DNA from US samples was isolated from dormant twigs. For leaf and twig samples, tissue (about 16 cm^2^ of leaf or a 6 cm section of twig with buds) was ground to a fine powder in liquid nitrogen using a mortar and pestle, then added to a tube of heated (55°C) CTAB extraction buffer and incubated for 4–6 h. Following incubation, DNA isolation was performed in 15 mL conical tubes using a phenol-chloroform extraction protocol, and DNA was precipitated in 0.2 M sodium chloride and isopropanol. After pelleting and resuspension of DNA in TE buffer, samples were cleaned using OneStep PCR Inhibitor Removal kits (Zymo Research, Irvine, CA, USA). Samples were quantified and quality assessed using a NanoDrop 8000 (Thermo-Fisher Scientific, Waltham, MA, USA) prior to pooling. Samples were pooled by source location at equimolar concentrations at a final volume of 200 uL and submitted for sequencing.

**Table 1:** *Castanea mollissima* DNA sample pools, with individuals (n) per sample site.

**Pool**	**Location**	**Origin**	**n^a^**	**Bases (quality clipped)**	**Estimated depth**	**Variant sites^b^**	**Sites with alt >0.2^c^**	**Average Depth^d^**
Y1	Yunnan- 26.013°N 101.0932°E	Forest	9	7,375,355,857	9.46	8,564,349	6,359,398	12.38
Y2	Yunnan- Fengqing County	Forest	10	11,619,019,171	14.89	11,431,073	9,371,773	17.56
S1	Shaanxi- Zhuque and Heihe Forests	Forest	13	7,344,176,568	9.42	13,492,467	8,279,766	9.46
S2	Shaanxi- Ningshan County	Forest	10	8,630,350,892	11.06	7,632,501	5,074,304	9.12
S3	Shaanxi- Liuba County	Forest	10	5,612,880,521	7.19	10,272,008	8,155,228	13.09
S4	Shaanxi-33.772° N 108.766° E	Orchard	10	9,890,960,153	12.68	17,086,140	12,681,410	13.96
GZ	Guizhou- 26.236° N 105.1676° E	Forest	10	8,594,920,262	11.02	10,046,443	7,949,407	14.10
HB	Hebei- 40.597° N 118.399° E	Orchard	10	6,175,704,628	7.92	7,796,678	5,479,063	11.03
BY	Beijing- Yanqing County	Orchard	10	5,240,552,948	6.72	6,844,594	4,301,595	9.03
ECC	Ohio, U.S.A.^e^	Orchard	12	3,498,422,441	4.49	4,939,429	2,079,207	7.10

a *Number of individuals in pool*.

b *Sites with a variant called in a given pool with read depth >6*.

c *Sites with an alternate allele of frequency at least 0.2*.

d *Average observed depth at variant sites numbered in the 8th column*.

e *Grown in Ohio, USA; derived from northern Chinese orchard cultivars*.

### DNA sequencing and assembly

Sequencing of 100 bp paired-end reads was carried out with an Illumina HiSeq 2500 (Illumina Inc., San Diego, CA, USA) at the Purdue Genomics Core Facility. Six genomic DNA pools (about 10 individuals each; Table 1) were sequenced per lane, with the goal of obtaining ~10x coverage per pool. Low-quality reads were filtered prior to assembly using Trimmomatic version 0.32 (Bolger et al., 2014).

Chloroplasts were sequenced by assembling short reads to the complete Chinese chestnut chloroplast reference sequence (Jansen et al., 2011). The 1.0 version of the Linkage Group A (LGA) pseudochromosome assembly and beta versions of the LGB-LGL assemblies (12 total) were obtained from Dr. John Carlson of Penn State University (Staton et al., 2014). Short reads were assembled to reference sequences using BWA, duplicates were flagged and alignment files sorted using Picard Tools, and SNPs were called using the HaplotypeCaller tool from the Genome Analysis ToolKit (GATK), with a polyploid value equal to the number of individuals in the pool. The Samtools mpileup tool was used to generate pileup-formatted SNP files for the orchard and wild sets of sample pools.

### Identification of regions under selection in the genome

Tajima's D and pi were calculated from mpileup files of orchard and wild assemblies using PoPoolation 2.0 (Kofler et al., 2011) over 10 kb windows for the entire genome. The difference in Tajima's D between orchard and wild pools was calculated and statistical significance tested using a permutation test encoded in a Perl script. Permutations were performed by assigning observed Tajima's D values within the orchard and wild pools of samples to a random base-pair interval of the genome and re-calculating the difference in Tajima's D between pools over the shuffled intervals. A *p*-value was assigned to each interval based on how many times a difference larger than the difference at that interval was observed in 1,000 shuffled genomes. Candidate loci for selection in orchard trees were intervals where the permuted *p*-value was less than 0.01. To reduce the false positive rate, we only considered for further analysis intervals where multiple consecutive 10 kb intervals showed significantly different (*p* < 0.01) values for Tajima's D and pi in orchard vs. wild trees, and/or a *p*-value less than 0.001. In addition, local false discovery rates for all 10 kb intervals were calculated using the qvalue package (Storey, 2002) in the R computing environment.

A second method for identifying regions in the genome under selection identified predicted gene intervals where the percent of SNPs that had one allele fixed was higher in one sample than in the other. The frequency of the major allele at SNP loci was averaged over all SNPs in a given predicted gene, and then the average major allele frequency was calculated for 10-gene intervals across the genome. Loci potentially under selection in orchard trees were identified based on the empirical distribution of the difference in the allele-frequency statistic over all predicted genes that had alignments to the UniProt database. A predicted gene was determined as potentially under selection if the difference in average major allele frequency between wild and orchard samples was greater than two standard deviations above the mean difference for all predicted genes in the genome. This method was used to identify genes under selection in orchard vs. wild trees, and also to identify loci with varying allele frequency among regional subpopulations of wild trees: northern (Shaanxi) vs. southern (Yunnan + Guizhou).

### Gene prediction and filtering

*De novo* gene prediction was carried out using AUGUSTUS (Stanke et al., 2006) with *Arabidopsis thaliana* as the training protein set and default settings. To assign a putative function to predicted genes, the predicted gene file (.gff) was converted to fasta (.fa) format and aligned to the UniProt protein database using the blastp function of the DIAMOND sequence aligner (Buchfink et al., 2015) using default settings. The top hit annotation on the UniProt website was used to assign a putative function to each gene.

To provide a measure of validation to this predicted gene set, publicly available cDNA contig files for American chestnut, Chinese chestnut, European chestnut, and Japanese chestnut were downloaded from http://www.hardwoodgenomics.org/transcriptomes. These were each aligned using the blastx function of DIAMOND, using default settings, to a database created using the predicted Chinese chestnut protein set output by AUGUSTUS. Transcripts were matched to the protein that provided the top hit from the predicted protein set; a predicted protein was only counted as having transcript support if it was the best alignment for at least one cDNA contig. This was carried out using a custom Perl script.

### Identification of chloroplast haplotypes

Chloroplast reads from whole-genome sequence data were assembled to the reference Chinese chestnut chloroplast genome using BWA and Picard Tools and SNPs were called using GATK with ploidy set equal to 10. A custom Perl script was developed that tallied the number of SNPs with a given alternate allele frequency (between 10 and 100%) in each pool as an approximation of the haplotype structure of the genome pools. For example, if a chloroplast haplotype with about 300 SNP variants vs. the reference was found in 30% of the samples from a pool, we expected to find about 300 SNP sites with 30% alternate allele frequency in that pool. Alternate chloroplast haplotypes were identified by peaks on a histogram of SNPs in allele frequency bins for each sample; the frequency of a haplotype was estimated by the bin where a “peak” occurred, and the haplotype identity estimated by the number of SNPs in an allele frequency bin (Figure S1). SNPs were compared with individual chloroplast sequences from Chinese chestnuts to determine whether haplotypes matched either of the two previously identified haplotypes.

### Validation of regions under selection

Whole-genome sequences of individual chestnuts were used to provide validation of regions under selection identified using pooled sequences. Tajima's D, nucleotide diversity, heterozygosity, and pi were calculated (VCFTools) using SNPs within exons of predicted genes for 18 Chinese chestnuts of southern Chinese and Korean provenance, as well as 2 American chestnuts, which represent non-domesticated trees. A negative value of Tajima's D, low values for pi, and proportion of heterozygous loci for a given predicted gene among individual orchard-derived Chinese chestnuts, were interpreted as support for a gene's selection during domestication. Synteny with other domesticated woody plants (peach, apple, and grapevine) was analyzed by aligning predicted proteins from domestication-related selective sweeps in peach (Cao et al., 2014), apple (Duan et al., 2017), and grape (Zhou et al., 2017) to predicted proteins from chestnut sweep regions. We considered there to be evidence of syntenic domestication regions if multiple chestnut proteins from a given regions were the best alignments for multiple proteins from a domestication region in another woody domestic plant. Correlation between the location of putative domestication selective sweeps and chestnut agronomic QTL was identified by aligning microsatellite and SNP markers from a QTL mapping experiment (Nishio et al., 2017) to the whole genome and calculating the distance (bp) between QTL-delimiting markers and putative domestication sweeps.

## Results

### Genome sequencing and assembly

Average estimated genome coverage for the pools sequenced was close to 1x per individual tree in a pool for most of the sequenced pools (Table 1) and was greater than 7x for all but two of the pools sequenced. The number of polymorphisms with alternate allele frequencies >0.2, which are less likely to result from sequencing errors, was highest in the Shaanxi orchard sample and lowest in the Beijing-derived orchard sample from Ohio (Table 2). The genomes of most of the orchard samples had fewer polymorphisms than wild trees.

**Table 2:** Notable regions under selection during chestnut domestication with statistical support and functional annotations.

**LG^a^**	**Start^b^**	**Gene^c^**	**Gene function potentially under selection^d^**	**D_o_^e^**	**D_w_^f^**	**p^g^**	**het_cm^h^**	**pi-cm^i^**	**pi-cd^j^**	**cd/cm^k^**	***F*_ST_^l^**
LGA	17560000	lga_g2116	1-aminocyclopropane-1-carboxylate oxidase	−1.57	0.31	0.006	0.19	0.0014	0.0015	1.11	0.76
**LGA**	**28940000**	**lga_g3565**	**Putative phytosulfokines PSK6**	−**1.65**	**1.23**	<0.001^*^	**0.14**	**0.0009**	**0.0037**	**4.26**	**0.44**
LGA	46000000	lga_g5798	Flowering time control protein FCA	−2.03	−0.47	0.020	0.00	0.0000	0.0005	483.33	0.81
LGA	46360000	lga_g5850	Alkane hydroxylase MAH1	−1.98	−0.05	0.005	0.05	0.0013	0.0032	2.54	0.92
LGA	53300000	lga_g6764	Transcription factor bHLH78	−1.25	0.44	0.013	0.11	0.0009	0.0016	1.73	0.82
LGA	53710000	lga_g6816	Late embryogenesis abundant protein LEA5	−1.70	−0.07	0.016	0.10	0.0001	0.0007	4.56	0.85
LGA	58690000	lga_g7476	Probable polygalacturonase ADPG2	−2.12	−0.47	0.015	0.10	0.0008	0.0014	1.66	0.64
LGA	72490000	lga_g9205	Anthocyanidin 3-O-glucosyltransferase 2	−1.59	0.07	0.019	0.10	0.0008	0.0017	2.08	0.89
LGA	104070000	lga_g13074	Transcription factor GATA-type	−1.86	0.13	0.003	0.07	0.0012	0.0022	1.83	0.86
LGB	3070000	lgb_g404	Homeobox-leucine zipper protein ATHB-14	−1.92	−0.08	0.011	0.12	0.0009	0.0012	1.30	0.74
LGB	7750000	lgb_g1007	Transcription factor bHLH147	−1.64	0.43	0.004	0.14	0.0031	0.0043	1.37	0.38
**LGB**	**8100000**	**lgb_g1054**	**Desiccation-related protein PCC13-62**	−**2.51**	**0.17**	<0.001^*^	**0.12**	**0.0030**	**0.0061**	**2.05**	**0.70**
LGB	8690000	lgb_g1139	ZF-BED domain protein RICESLEEPER 1	−1.71	0.17	0.009	0.20	0.0012	0.0018	1.50	0.81
**LGB**	**19610000**	**lgb_g2523**	**Sucrose synthase 2**	−**1.96**	**0.44**	**<0.001**	**0.16**	**0.0022**	**0.0045**	**2.04**	**0.46**
LGC	6500000	lgc_g807	Cytochrome P450 CYP71D312	−1.65	0.07	0.013	0.07	0.0019	0.0125	6.61	0.82
LGC	21310000	lgc_g2594	Reticuline oxidase	−1.68	0.56	< 0.001	0.07	0.0008	0.0024	2.84	0.86
LGC	30360000	lgc_g3816	Histone-lysine N-methyltransferase SUVH4	−1.61	0.27	0.007	0.23	0.0012	0.0008	0.72	0.68
LGC	49350000	lgc_g6157	1-aminocyclopropane-1-carboxylate oxidase 4	−1.52	0.15	0.015	0.04	0.0001	0.0017	17.21	0.91
LGC	50850000	lgc_g6330	Transcription factor RAX2	−1.53	0.49	0.003	0.02	0.0005	0.0029	5.90	0.97
LGD	7620000	lgd_g1017	SHOOT GRAVITROPISM 5, IDD15	−1.44	0.33	0.009	0.10	0.0002	0.0008	3.34	0.58
LGD	18020000	lgd_g2376	Major allergens Pru1	−2.51	0.17	< 0.001	0.12	0.0006	0.0033	5.34	0.88
LGE	50780000	lge_g6427	POLLENLESS 3, MS5	−1.71	0.39	0.001	0.04	0.0014	0.0116	8.25	0.63
LGG	13830000	lgg_g2955	Peroxisome biogenesis protein 1	−1.87	0.09	0.003	0.00	0.0001	0.0027	23.02	0.94
LGG	24830000	lgg_g3130	LEA protein D-29	−1.29	0.52	0.007	0.15	0.0011	0.0012	1.07	0.79
LGG	49050000	lgg_g6369	Syntaxin-132, isocitrate lyase	−2.08	−0.29	0.008	0.13	0.0008	0.0015	1.84	0.73
LGH	780000	lgh_g105	Ras-related protein RAA4b	−2.23	0.35	< 0.001	0.17	0.0025	0.0046	1.88	0.67
LGI	10470000	lgi_g1363	Syntaxin-132	−1.59	0.34	0.008	0.19	0.0010	0.0006	0.58	0.68
LGI	33370000	lgi_g4214	FT (Flowering Time) -interacting protein 1	−2.08	0.33	< 0.001	0.10	0.0017	0.0023	1.36	0.77
LGI	40500000	lgi_g5153	Peroxidase 24	−1.94	0.33	0.001	0.10	0.0017	0.0073	4.39	0.66
LGJ	16440000	lgj_g2112	Transcription factor VRN1	−2.09	0.03	0.002	0.23	0.0024	0.0043	1.76	0.52
LGJ	16890000	lgj_g2169	Universal stress protein PHOS34	−1.78	0.28	0.003	0.17	0.0019	0.0030	1.63	0.39
**LGK**	**25200000**	**lgk_g3168**	**Floral homeotic protein AGAMOUS**	−**2.02**	**0.10**	**0.001**	**0.19**	**0.0010**	**0.0026**	**2.55**	**0.72**
LGL	38090000	lgl_g4810	1-aminocyclopropane-1-carboxylate synthase 7	−2.08	0.25	< 0.001	0.24	0.0059	0.0038	0.65	0.50

*Bolded entries indicate intervals with local false discovery rates < 0.25, calculated by qvalue (Storey, 2002)*.

a Linkage group and

b *starting position of sweep region in pseudochromosome draft assembly (Staton et al., 2014)*;

c *Predicted gene selected from sweep based on annotation and statistical significance*;

d *Annotation of selected gene*;

e Tajima's D for orchard and

f *wild Chinese chestnut pools across the sweep interval*;

g *Permutation-derived p value for sweep*;

h *Average heterozygosity for the selected gene in an independent sample of 12 Chinese individual chestnut genomes*;

i Nucleotide diversity among Castanea mollissima (Cm) and

j *Castanea dentata (Cd) for the selected gene*;

k *Factor by which π was greater in wild Cd than in domesticated Cm*;

l *F_ST_ calculated between Cm and Cd using SNPs in the selected gene interval*.

* *Local false discovery rate < 0.05*.

### Regions under selection

Tajima's D, used as a measure of selection pressure, was on average lower in orchard pools (−0.64) than in wild (−0.50). Using the Tajima's D and pi outlier method, >100 intervals were significantly different between wild and orchard trees, as determined by permutation tests with a significance cutoff of *p* < 0.01 for a given 10,000 base-pair interval (Table S1); several intervals with large differences in Tajima's D were chosen for further annotation (Table 1). The major allele frequency across predicted gene sequences was slightly higher for orchard chestnuts (0.693) than for wild chestnuts (0.685). Using the allele frequency method to identify regions under selection, the standard deviation of the difference in major allele frequency between orchard and wild pools was used to identify outliers (cutoff: >3 standard deviations greater than mean difference for orchard vs. wild and >2 sd for regional differences), which led to the identification of approximately 25 candidate loci for domestication and 15 for regional genetic differences (Tables 3, **5**, Table S3). The identified candidate loci contained predicted flowering-time genes, genes involved in the synthesis of ethylene, genes influencing male fertility, cell wall structure, secondary metabolites, and disease resistance (Tables S2, S4). Candidate loci under selection showed lower-than average heterozygosity and nucleotide diversity in Chinese chestnut and, in many cases, greater nucleotide diversity in American chestnut than Chinese chestnut (Table S5). Several predicted proteins in putative selective sweeps of chestnut were likely homologs of predicted proteins in selective sweep regions of peach, apple, and grapevine (Tables S2, S4, S6); in total, 11 of the identified sweep regions in chestnut showed evidence of synteny with domestication candidate regions with at least one other woody plant.

**Table 3:** Additional regions under selection due to domestication and regional climatic variation identified by allelic fixation at SNPs.

**LG^a^**	**Start^b^**	**Gene^c^**	**Annotation^d^**	**O^e^**	**W^f^**	**Sd^g^**	**Het-*Cm*^h^**	**π-*Cm*^i^**	**π-*Cd*^j^**	***Cd*/*Cm*^k^**	***F*_ST_^l^**
LGA	50030000	lga_g6327	THOC6_ARATH THO complex subunit, ABA signaling	0.93	0.63	>3	0.05	0.0004	0.0019	4.47	0.82
LGA	65859000	lga_g8373	RPE_SOLTU Ribulose-phosphate epimerase	0.86	0.56	>3	0.04	0.0006	0.0021	3.53	0.93
LGC	31478000	lgc_g3960	PP14_ARATH Serine/threonine protein phosphatase	0.86	0.64	>3	0.04	0.0006	0.0019	3.27	0.72
LGE	25481000	lge_g3428	MYBF_ARATH, transcription factor	0.87	0.71	>3	0.08	0.0007	0.0020	2.87	0.67
LGE	29262000	lge_g3727	CE101_ARATH lectin receptor kinase	0.93	0.66	>3	0.07	0.0003	0.0057	18.48	0.82
LGE	44249000	lge_g5605	DRE2D_ARATH Dehydration-responsive element	0.92	0.61	>5	0.03	0.0002	0.0015	6.67	0.85
LGF	16717000	lgf_g2053	ALsF1_ARATH Aldehyde dehydrogenase	0.84	0.60	>3	0.09	0.0002	0.0009	5.13	0.86
LGF	27956000	lgf_g3433	SAUR32_ARATH Auxin responsive element	0.84	0.63	>3	0.09	0.0004	0.0007	1.81	0.90
LGG	8420000	lgg_g5871	CDPK_SOYBN calcium-dependent protein kinase SK5	0.96	0.64	>3	0.11	0.0015	0.0025	1.64	0.49
LGG	2054000	lgg_g2558	GONS1_ARATH GDP-mannose transporter	0.90	0.68	>3	0.08	0.0006	0.0016	2.63	0.86
LGG	23410000	lgg_g1699	LEA34_GOSHI late-embryogenesis-abundant protein	0.85	0.59	>3	0.20	0.0004	0.0015	3.82	0.68
LGG	33377000	lgg_g4266	MEE14_ARATH CCG-binding AGAMOUS interactor	0.92	0.64	>3	0.11	0.0009	0.0029	3.16	0.78

a Linkage group and

b *starting position of 10 kb sweep region in pseudochromosome draft assembly (Staton et al., 2014)*;

c *Predicted gene selected from sweep based on annotation and statistical significance*;

d *Annotation of selected gene*;

e Average major allele frequency for SNPs in orchard and

f *wild Chinese chestnut pools across the sweep interval (10 kb)*;

g *Permutation-derived p value for sweep*;

h *Average heterozygosity for the selected gene in an independent sample of 12 individual Chinese chestnut genomes*;

i Nucleotide diversity among orchard-derived Chinese chestnut, Castanea mollissima (Cm) and American chestnut

j *Castanea dentata (Cd) for the selected gene*;

k *Factor by which π was greater in wild Cd than in domesticated Cm*;

l *F_ST_ calculated between Cm and Cd using SNPs in the selected gene interval*.

### Chloroplast haplotypes

The reference chloroplast haplotype was found at its highest frequency in one Yunnan sample (100%) and the Guizhou sample (~60%), and at its lowest frequencies in the Hebei and ECC orchard samples (~10%) (Figure S2). One alternate haplotype was present in the Guizhou (~40%), Hebei (~90%), ECC (90%), Beijing (~20%), and Shaanxi-3 (~90%) pooled samples (Figures S2, S3). This haplotype, which had about 260 SNP polymorphisms different from the reference, was found to be the same as the (non-reference) *C. mollissima* chloroplast of “Clapper” (LaBonte et al., in preparation). Other polymorphic sites did not correspond to the “Clapper” haplotype, so additional haplotypes must have been present in some of the sampled populations. A highly divergent (1000+ SNPs different from reference) haplotype appears to be present at relatively low frequency in the Shaanxi-1, Shaanxi-4, and Yunnan-2 samples (Figure S3), and an additional haplotype with low divergence from the reference, about 75 SNPs, appears to be present in the Shaanxi-1 sample (Figure S2).

## Discussion

### Chloroplast assemblies and genetic diversity

Genotyping of pooled chloroplasts indicated the presence of several haplotypes not identified in a previous survey of *Castanea mollissima* chloroplast genome assemblies (LaBonte et al., in preparation). Other than the reference haplotype, the most common and widely-distributed haplotype was variant at ~250 sites and was most abundant in northern Chinese orchard samples, but is not particularly common in American orchard germplasm. The reference haplotype was most abundant in Southern Chinese wild samples; its abundance in the US population of Chinese chestnut supports a southern origin for most US chestnut germplasm. The Shaanxi orchard chestnut sample's chloroplast genotype profile resembled the wild Shaanxi-1 chloroplast profile more than it did the other orchard samples, which indicated that admixture between local wild populations and orchard trees is probably extensive in cultivated Chinese chestnut. The chloroplast haplotype shared by “Clapper” and two of the orchard pools (Hebei and ECC) was also found at high frequency in the Shaanxi-3 wild sample. The diversity of chloroplast haplotypes evident in the three wild Shaanxi samples supports earlier findings that the Qinling (=Dabashan) range in Shaanxi province represents a center of genetic diversity for *C. mollissima* (Cheng et al., 2012; Liu et al., 2013a). More sampling of whole chloroplast genomes is needed determine the true number of unique haplotypes, especially in the Shaanxi and Yunnan chestnut populations, where the strongest evidence for diversity was observed.

**Figure 2:**
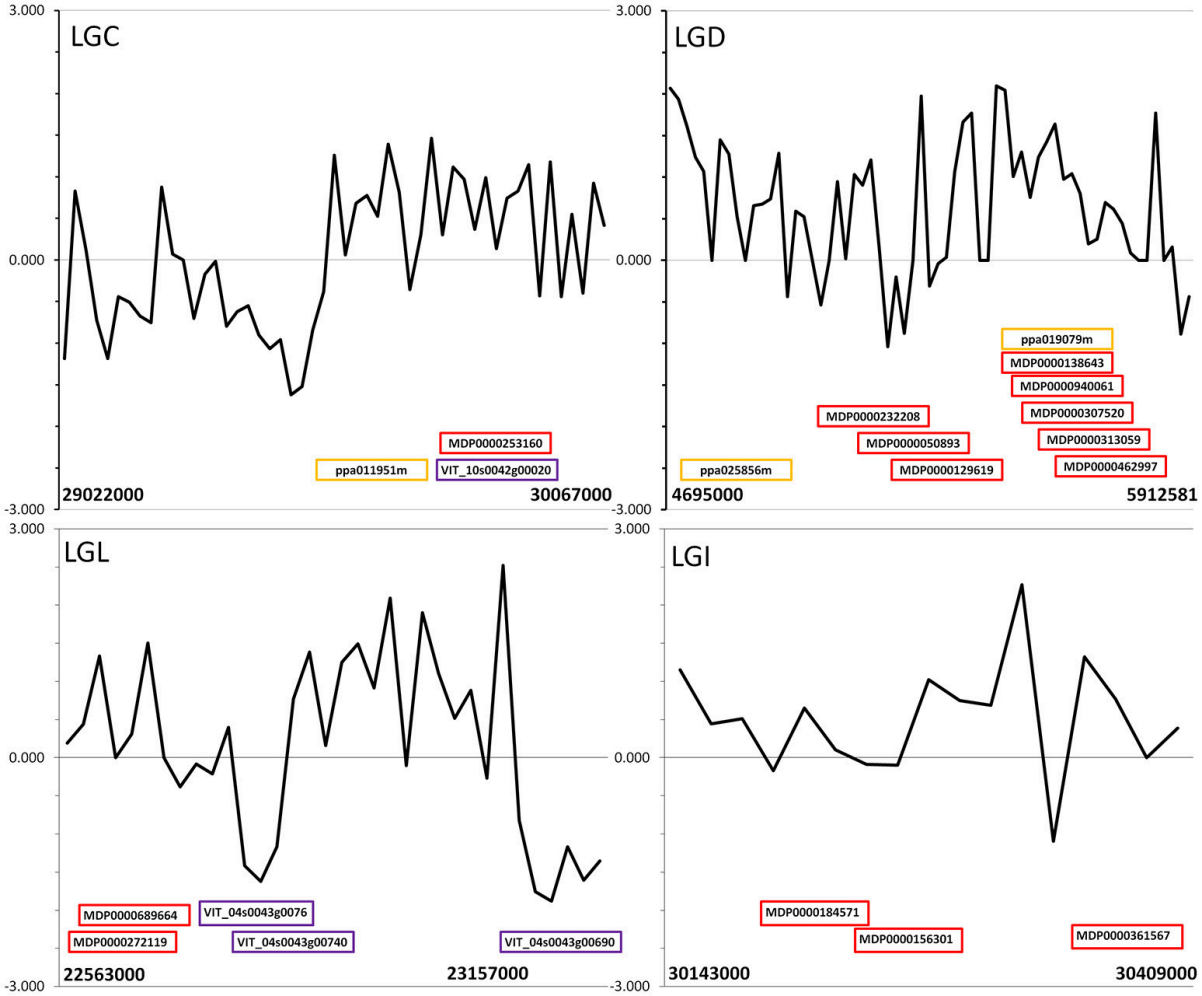
Tajima's D statistic in an independent sample of 8 orchard-derived chestnut whole-genome sequences, graphed over putative selective sweeps on LGC, LGD, LGL, and LGI of the Chinese chestnut genome identified using pooled whole-genome data. Approximate locations of predicted chestnut genes that were the best alignment for genes in domestication-associated selective sweeps of apple (red), grape (purple) and peach (orange) are labeled with the name of the aligned apple, grape, or peach gene.

Previous studies of genetic diversity in wild and orchard Chinese chestnuts found relatively high genetic diversity maintained in orchard trees (Pereira-Lorenzo et al., 2016). It appears to be the case that, like other perennial woody food plants (Cornille et al., 2012) the overall reduction in genetic diversity in chestnut due to domestication has been limited. Despite this, the number of 50–100 kb regions in the genome where orchard trees had low genetic diversity relative to wild trees was about 10 times larger than the number of regions where orchard trees had higher nucleotide diversity than wild trees. It is possible that lower genome coverage in orchard samples led to underestimates of heterozygosity. The same minimum coverage filter (8x) was implemented for the SNP sets from orchard and wild pools during data analysis, however, to minimize bias due to lower coverage of orchard tree genomes. Using individual whole-genome SNP data from 17 orchard-grown Chinese chestnuts and two American chestnuts, we were able to identify several loci that showed strong evidence of low genetic diversity both in orchard pools and in orchard chestnuts relative to the non-domesticated American chestnut. These predicted genes (Tables 2, 3; also highlighted in Tables S2, S4) we consider the best candidates for chestnut domestication. Several putative chestnut sweeps (on LGA, LGC, LGD, LGI, and LGL) contained multiple predicted genes with >60% amino acid identity to predicted genes from sweeps in apple (Duan et al., 2017), peach (Cao et al., 2014), and grape (Zhou et al., 2017) (Figure 2, Table 4, Table S6), indicating that some syntenic loci have likely been selected in multiple domesticated woody plants.

**Table 4:** Evidence of synteny between chestnut domestication candidate loci and domestication-associated chromosomal regions in other woody plant crops.

**Chestnut domestication locus**	**Aligned proteins^a^: Apple**	**Aligned proteins: Peach**	**Aligned proteins: Grape**
LGA:50030000	0	0	3
LGA:65859000	1	0	3
LGB:**19610000**	1	0	0
LGC: 6505000	1	1	1
LGC: 30360000	2	0	0
LGD: 5350000	9	1	0
LGE: 25481000	0	2	0
LGF: 27956000	2	0	0
LGI:27225000	1	2	0
LGI:33385000	3	0	0
LGL:23280000	2	0	3

a *Number of predicted proteins from a domestication region in apple, peach, or grape that were the best alignment for a protein in the indicated chestnut selective sweep region in chestnut, in an alignment of all chestnut proteins vs. all apple, peach, and grape proteins*.

### Functional annotation of regions under selection in chestnut domestication

Domestication candidate loci with the strongest statistical evidence, considering permutation tests, local false discovery rate calculations, and nucleotide diversity in independent whole-genome SNP datasets from orchard-derived Chinese chestnuts (Table 3) included several predicted genes with annotations that indicate a potential role in chestnut domestication. One locus on LGA included a predicted gene similar to a putative phytosulfokines 6 protein from Arabidopsis, which is a growth regulator active during embryogenesis (Matsubayashi et al., 2006). Additional highly significant loci included a dessication-related protein and a sucrose-synthase (Angeles-Nunez and Tiessen, 2010) like protein on LGB; the latter protein is highly similar (90.9% peptide identity) to a domestication candidate (MDP0000859573) on chromosome 13 of apple (Duan et al., 2017).

Several additional loci contained predicted gene annotations pointing to potential roles in chestnut domestication. One, also on LGA, was similar to anthocyanidin 3-O-glucosyltransferase 2 (LGA) of wine grapes (*Vitis vinifera*), which is responsible for the synthesis of red wine pigments (Ford et al., 1998). The existence of Chinese chestnut cultivars with enhanced red coloration in their leaves and twigs (Junhao et al., 2000) indicates that increased anthocyanin production was selected *for* during domestication.

Genes that regulate flower development and timing are among the most frequently identified in selective sweeps related to plant domestication (e.g., Kaga et al., 2008; Schmutz et al., 2014). Predicted genes similar to known flowering-time regulatory genes were found at several putative selective sweep loci. Putative domestication sweep regions included predicted genes similar to FLOWERING LOCUS C (FLC), a MADS-box protein that functions as major floral development repressor (Choi et al., 2009); FTIP1 of *Arabidopsis*, which exports the essential flowering control protein FLOWERING TIME (FT) into phloem sieve elements (Liu et al., 2012), POLLENLESS, a male fertility locus (Glover et al., 1998), AGAMOUS, which controls organ identity in developing flowers (Drews et al., 1991), and SUVH4 which suppresses a transcriptional regulator (Jackson et al., 2002) involved in female floral development (Sakai et al., 1995). The FLOWERING LOCUS C homolog showed a particularly strong signature of selection in the 17 whole-genome sequences we obtained from orchard-derived Chinese chestnuts (Tables S2, S4, S6) vs. non-domesticated American chestnut. The POLLENLESS_like gene is intriguing because a short-catkin mutation of Chinese chestnut has previously been identified (Feng et al., 2011), and some *Castanea sativa* cultivars with exceptionally large nuts (“marron” types) actually produce astaminate catkins that are sterile (Pereira-Lorenzo et al., 2006, 2016).

A number of the predicted genes in the regions with signatures of selection in orchard trees were similar to genes in model plants that are involved in the regulation of plant development and cell wall modification: a shoot gravitropism regulator (SGR5 or IDD15) of *Arabidopsis*, which regulates branch orientation (Cui et al., 2013) and starch levels (Tanimoto et al., 2008), a cell-number regulation enzyme of maize (LGC) that affects plant organ size and is homologous to a major fruit weight QTL gene in tomato (Guo et al., 2010), *Arabidopsis* RABA4B, a Golgi-network trafficking regulatory protein that may involved in the secretion of cell wall components (Preuss et al., 2004), and a polygalacturonase similar to ADPG2 in *Arabidopsis*, which is involved in pod shattering (González-Carranza et al., 2007; Ogawa et al., 2009) Modification of cell walls is a major part of fruit ripening, which is why polygalacturonases, cellulases, and other cell-wall enzymes have been discovered in selective sweeps in the genomes of domesticated tomato and pepper (Paran and van der Knaap, 2007). The IDD15-like locus may correspond to a Japanese chestnut nut weight QTL (Nishio et al., 2017), and the RABA4-like and polygalacturonase-containing loci correspond closely to QTL identified for harvest time in Japanese chestnut (Nishio et al., 2017).

Management of environmental stresses—heat and drought tolerance, as well as insect pests and fungal diseases—is currently a goal of chestnut breeding programs in China (Gaoping et al., 2001). It is likely that stress tolerance has been under selection throughout Chinese chestnut's history of cultivation. Management of disease and environmental stress was the inferred role of several predicted genes within the putative domestication intervals: one similar to the ethylene-responsive transcription factor ERF3; late-embryogenesis-abundant (LEA) proteins from orange (*Citrus aurantium var. chinensis*) and cotton (*Gossypium hirsutum*), which are believed to have a role in desiccation tolerance of seeds and vegetative tissues (Battaglia et al., 2008); homeobox-leucine zipper transcription factor proteins ATHB-6, involved in water deficit responses (Söederman et al., 1999); and a predicted peroxidase similar to a protein in Arabidopsis which is upregulated in response to cold (Fowler and Thomashow, 2002).

Phytohorome metabolism, and transcription factors that regulate plant development, are commonly associated with domestication-related selective sweeps, such as the bHLH and MYB-family transcription factors identified in domestication sweep regions of the genomes of peach (Cao et al., 2014) and apple (Khan et al., 2014; Duan et al., 2017), as well as other plants (e.g., Schmutz et al., 2014). Several MYB- and bHLH-type transcription factors were found in regions that showed evidence of strong selection in the genomes of orchard chestnuts. One basic helix-loop-helix (bHLH)—type transcription factor in a sweep region may be a homolog to the *Arabidopsis* bHLH78 transcription factor, which promotes the expression of the Flowering Time gene and therefore is involved in the initiation of flowering (Liu et al., 2013b). One putative selective sweep (LGD) containing a predicted MYB-type transcription factor that corresponded to a QTL (Nishio et al., 2017) for bur number/tree in Japanese chestnut. Two individual selective sweeps on different linkage groups (LGA, LGC) contained predicted genes that were similar to 1-aminocyclopropane-1-carboxylate oxidase genes from *Arabidopsis* and a third (LGL) contained one that was similar to 1-aminocyclopropane-1-carboxylate synthase. The products of these genes together regulate the production and degradation of the plant hormone ethylene (Yamagami et al., 2003; Qin et al., 2007). It is not clear, however, whether these ethylene-related genes influence nut ripening, stress response, or other processes.

Most loci with regional differences in allele frequency were closer to fixation in the southern samples of wild trees (Yunnan and Guizhou) than in the northern sample (Shaanxi), with the exception of one interval on LGE that contained a predicted gene similar to cinnamoyl alcohol dehydrogenase from *Eucalyptus botryoides*, and another on LGH that was similar to a senescence-associated protein from *Arabidopsis* (Table 5). The locus on LGE is intriguing because it may correspond to a QTL for resistance to *Phytophthora cinammomi* resistance in hybrids of Chinese and American chestnut (Olukolu et al., 2012). It is possible that more alleles for this gene are present in southern Chinese populations of chestnut to combat variable races of *P. cinnammomi*, which thrive in warm climates. Several other genes in regions with differentiated allele frequencies among regional subpopulations included several lignin-synthesis genes, and a DRE1B-type gene, all of which are probably involved in cold-tolerance. Interestingly, one predicted gene that had decreased allele frequency in southern China was similar to a transcription factor in *Arabidopsis* that controls trichome density (Schnellmann et al., 2002). Increased trichome density could be favorable in warmer climates where water loss is more severe during hot weather.

**Table 5:** Putative loci differentially selected among northern and southern samples of wild Chinese chestnut, identified by comparing allele frequencies among pools of chestnut, with annotations based on the best UniProt alignments of predicted genes.

**LG^a^**	**Start**	**End**	**N^b^**	**S^c^**	**Sd^d^**	**Predicted gene^e^**
LGA	72907000	72988000	0.56	087	>2	C94A2_VICSA: cytochrome P450, fatty acid oxidation
LGA	79800000	79880000	0.67	0.95	>3	Y2060_ARATH: BTB/POZ domain ubiquination protein
LGA	80300000	80330000	0.61	0.89	>3	SD25_ARATH: protein kinase
LGA	82239000	82355000	0.65	0.99	>4	PLY19_ARATH: pectate lyase 19
LGB	15342000	15410000	0.64	0.91	>3	E134_MAIZE: endo-1,3;1,4-beta-D-glucanase
LGB^*^	6540000	6678000	0.71	0.95	>3	SEOB_ARATH^*^, sieve-element occlusion protein
LGC	48510000	48811632	0.66	0.83	>2	SIB1_ARATH: sigma binding factor, pathogen defense
LGC^*^	50000000	50186000	0.69	0.95	>3	CNR2_MAIZE^*^, cell-number regulator
LGC	53870000	53947000	0.57	0.86	>3	PP413_ARATH: pentatricopeptide repeat-containing protein
LGE	16600000	16700000	0.67	1.00	>4	CCR1_ARATH: cinnamoyl-CoA reductase, lignin synthesis; *Phytophthora cinammomi* resistance?
LGG	43890000	43990000	0.70	0.99	>3	HMDH1_GOSHI: isoprenoid precursor (mevalonate) synthesis
LGG	48970000	49040000	0.61	0.88	>3	CPC_ARATH: trichome development transcription factor
LGI	4295000	4336000	0.62	0.89	>4	SILD_FORIN, ILR1_ARATH: lignin biosynthesis
LGL	58890000	59190000	0.69	0.97	>4	ERF25_ARATH, DRE1B_ARATH: cold tolerance
LGE	34000000	34100000	0.91	0.65	>3	CADH_EUCBO: cinnamoyl alcohol dehydrogenase, lignin synthesis
LGH	18500000	18547000	0.81	0.62	>3	SAG13_ARATH: senescence-associated protein

*Gray shading indicates loci with evidence of directional selection in northern Chinese samples*.

a *Average major allele frequency in northern Chinese wild trees for the given interval of 10 predicted genes*;

b *Average major allele frequency in southern Chinese wild trees for the given interval of 10 predicted genes*;

c *Standard deviations greater than the average difference in major allele frequency between orchard and wild pools*.

d *Standard deviations from mean difference in allele frequency between northern and southern pools*;

e *Annotation of predicted chestnut gene (AUGUSTUS) based on alignment to the UniProtKB/Swiss-Prot database*.

* *Also identified as putative domestication loci due to low Tajima's D-value in orchard samples*.

## Conclusions

Our study provides a first glimpse into the complex pathways of selection by which humans transformed a forest tree into a reliable food crop, but also has practical importance for chestnut improvement. For breeders who are interested in improving Chinese chestnut for increased nut production or nut size, genes that were selected during domestication to promote heavier fruiting, such as the male-sterility genes identified here, could be a pathway to trees with shorter catkins and more female flowers. Many of the genes potentially involved in cuticular wax synthesis, stress tolerance, and synthesis of secondary compounds could be used for improving storage quality and pest resistance of chestnuts. For breeders who are interested in transferring disease resistance from Chinese chestnut into other species, genes involved in orchard-type crown architecture might be desirable or undesirable, depending on the phenotypic goals of the program. Conversely, some of the genes identified in these sweep regions may be desirable for improving the resistance of other chestnut species to pests like Asian gall wasp and *Phytophthora* root rot. More research is needed to determine the actual phenotypic effects of the gene loci identified here, but our results provide a glimpse of selective pressure on the chestnut genome during the tree's domestication, and a rough sketch of a map for future genomics-assisted chestnut improvement.

## Data statement

All sequence data associated with this project is stored in a sequence read archive (SRA) on the GenBank website with accession number (PENDING). Custom Perl scripts (e.g., the permutation test) used in this research are available upon request from the corresponding author.

## Author contributions

NL carried out DNA extraction, sequencing, and analysis as part of his doctoral research. PZ supervised collection of Chinese chestnut samples from wild and orchard populations. As NRL's doctoral advisor KW provided guidance for the research.

### Conflict of interest statement

The authors declare that the research was conducted in the absence of any commercial or financial relationships that could be construed as a potential conflict of interest.
