# Supplementary material for: Signatures of Selection in the Genomes of Chinese Chestnut (Castanea mollissima Blume): The Roots of Nut Tree Domestication
Source: Front Plant Sci. 2018 Jun 25;9:810. doi: 10.3389/fpls.2018.00810 (PMC6026767; doi:10.3389/fpls.2018.00810)
Supplement: Supplementary file 2 [file Image_1.pdf]

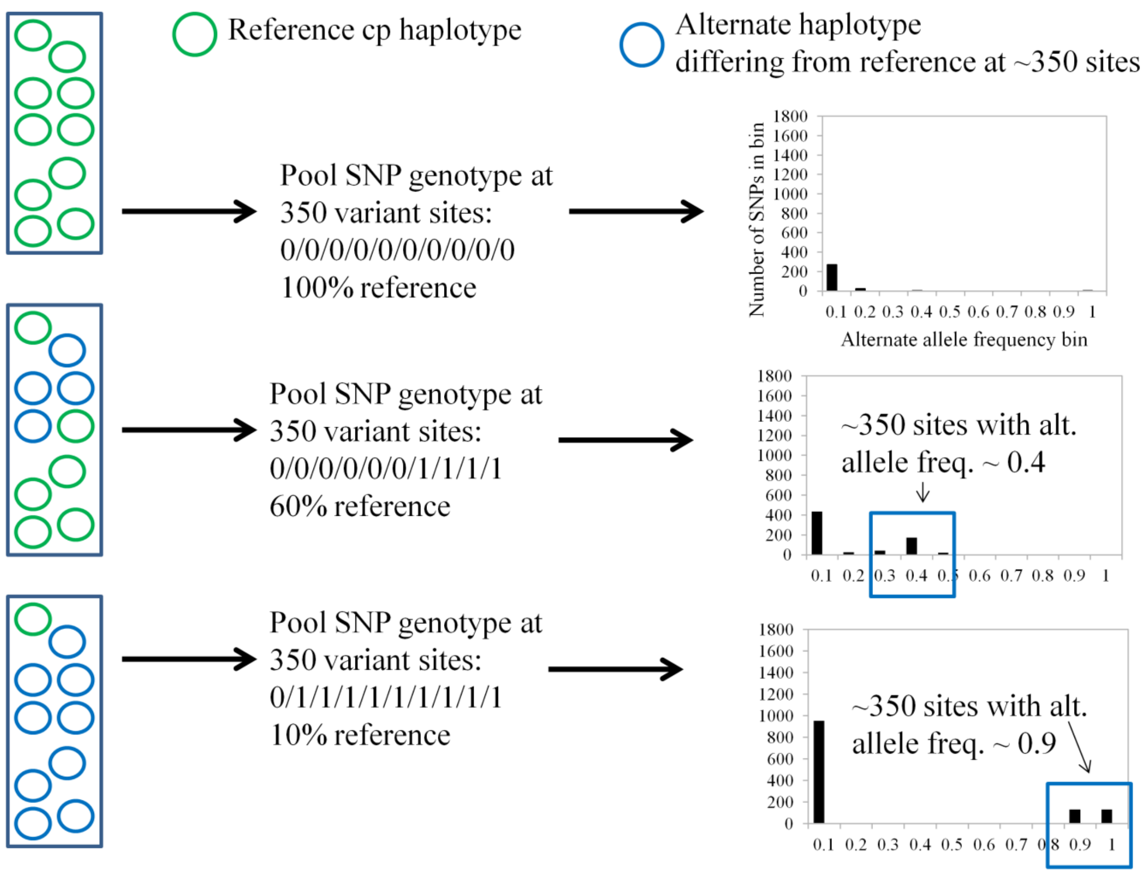

Figure S1. Depiction of alternate chloroplast haplotype frequency estimation from pooled samples. When SNP loci in the chloroplast are binned by the frequency of alternate alleles, the number of SNPs at which a given alternate haplotype differs from the reference (Y axis of histogram) and the frequency of the alternate genotype (X axis of histogram) can be estimated.

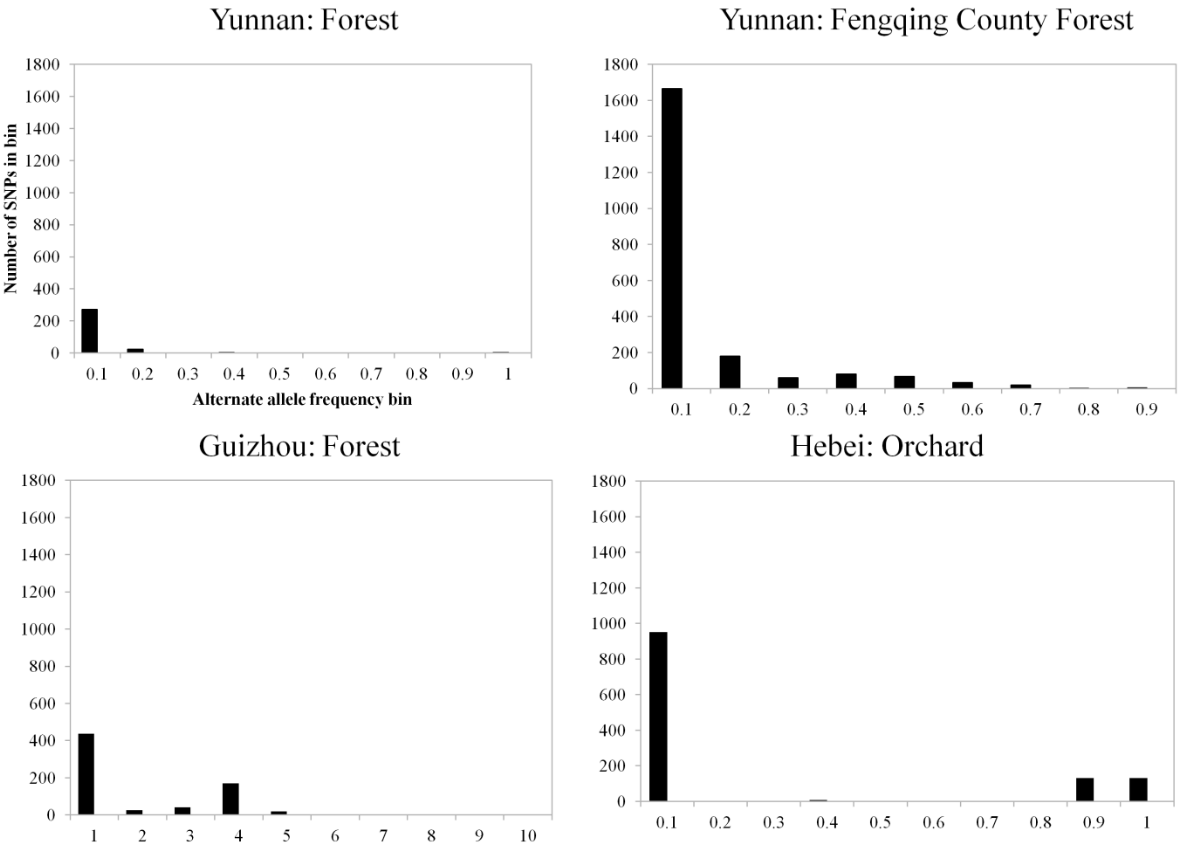

Figure S2. Chloroplast SNP alternate allele frequency histograms, with alternate allele frequency on the X axis and number of SNPS with a given alternate allele frequency on the Y axis, for pooled chestnut samples from southern (Yunnan, Guizhou) and northern (Hebei) China. An alternate chloroplast occurs at high frequency in the Hebei sample, while the reference chloroplast dominates one Yunnan sample and several haplotypes may be present in the Yunnan-Fengqing County sample.

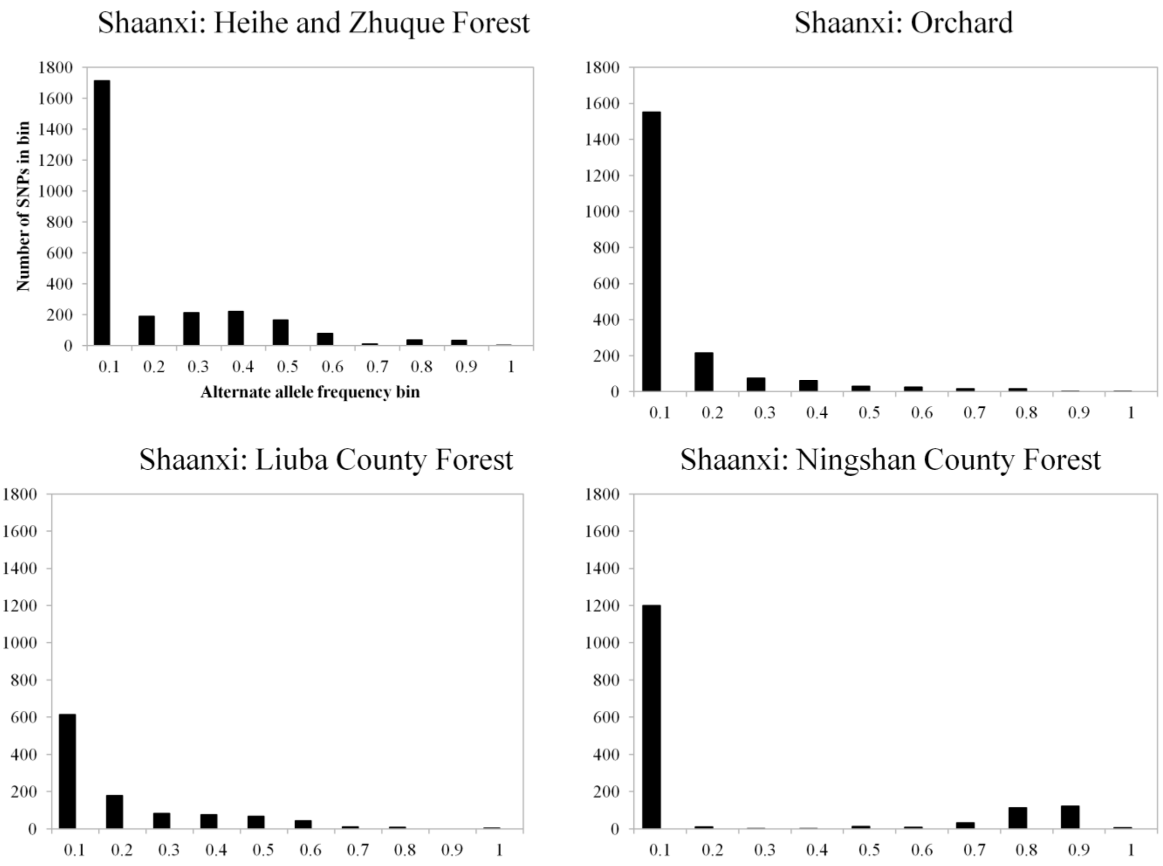

Figure S3. Chloroplast SNP alternate allele frequency histograms, with alternate allele frequency on the X axis and number of SNPS with a given alternate allele frequency on the Y axis, for pooled chestnut samples from Shaanxi Province in northwestern China. At least two haplotypes (peaks at frequency = 0.4 and = 0.8 are evident in the Heihe/Zhuque forest sample.
